# Supplementary material for: Heat adaptation of phage T7 under an extended genetic code
Source: Virus Evol. 2021 Dec 1;7(2):veab100. doi: 10.1093/ve/veab100 (PMC8923235; doi:10.1093/ve/veab100)
Supplement: veab100_Supp [file veab100_supp.zip › Sequencing_Analysis.zip!/Sequencing_Analysis/A1/evidence/SNP_2.html]

BRESEQ :: Evidence


| Predicted mutation | | | | | | |
| --- | --- | --- | --- | --- | --- | --- |
| evidence | seq id | position | mutation | annotation | gene | description |
| RA | NC\_001604.1 | 15,094 | G→T | intergenic (–/–) | – / – | –/– |

| Read alignment evidence... | | | | | | | | | | | |
| --- | --- | --- | --- | --- | --- | --- | --- | --- | --- | --- | --- |
|  | seq id | position | | ref | new | freq | score (cons/poly) | reads | annotation | genes | product |
| \* | NC\_001604.1 | 15,094 | 0 | G | T | 98.9% | 6518.2 / NA | 2991 | intergenic (–/–) | *–/–* | –/– |
| Reads supporting (aligned to +/- strand):  **ref** base G (9/24);  **new** base T (1601/1343);  **total** (1616/1375) | | | | | | | | | | | |
| Fisher's exact test for biased strand distribution *p*-value = 2.37e-03 | | | | | | | | | | | |
| Kolmogorov-Smirnov test that lower quality scores support variant *p*-value = 1.00e+00 | | | | | | | | | | | |

Only 100 of 3001 total aligned reads displayed.  

`AGAGCAGGGTGAAGAATACGTTGACGGAATGGAGTGGTGGAACTTCAACGAAGAGATGATGGACTATAACGTTCAGGACGTTGTGGTAACTAAAGCTCTCCTTGAGAAGCTACTCTCTGACAAACATTACTTCCCTCCTGAGATTGACTTTACGGACGTAGGATACACTACGTTCTGGTCAGAATCCCTTGAGGCCGTTGACATTGAACATCGTGCTGCATGGCTGCTCGCTAAACAAGAGCGCAACGGGTTCCCGTTTGACACAAAAGCAATCGAAGAGTTGTACGTAGAGTTAGCTGCTCGCCGCTCTGAGTTGCTCCGTAAATTGACCGAAACGTTCGGCTCGTGGTATCAGCCTAAAGGTGGCACTGAGATGTTCTGCCATCCGCGAACAGGTAAGCCACTACCTAAATACCCTCGCATTAAGACACCTAAAGTTGGTGGTATCTTTAAGAAGCCTAAGAACAAGGCACAGCGAGAAGGCCGTGAGCCTTGCGAACTTGATACCCGCGAGTACGTTGCTGGTGCTCCTTACACCCCAGTTGAACATGTTGTGTTTAACCCTTCGTCTCGTGACCACATTCAGAAGAAACTCC  >  NC_001604.1/14796‑15391`  

                                                                                                                                                                                                                                                                                                          `|`                                                                                                                                                                                                                                                                                                           
      `gggTGAAGAATACGTTGACGGAATGGAGTGGTGGAACTTCAACGAAGAGATGATGGACTATAACGTTCAGGACGTTGTGGTAACTAAAGCTCTCCTTGAGAAGCTACTCTCTGACAAACATTACTTCCCTCCTGAGATTGACTTTACGGACGTAGGATACACTACGTTCTGGTCAGAATCCCTTGAGGCCGTTGACATTGAACATCGTGCTGCATGGCTGCTCGCTAAACAAGAGCGCAACGGGTTCCCGTTTGACACAAAAGCAATCGAAGAGTTGTACGTAGAGTTAGCTTCTcgccgc                                                                                                                                                                                                                                                                                                   >  1:72072/1‑301 (MQ=255)`  
       `ggTGAAGACTACGTTGACGGAATGGAGTGGTGGAACTTCAACGAAGAGATGATGGACTATAACGTTCAGGACGTTGTGGTAACTAAAGCTCTCCTTGAGAAGCTACTCTCTGACAAACATTACTTCCCTCCTGAGATTGACTTTACGGACGTAGGATCCACTACGTTCTGGTCAGAATCCCTTGAGGCCGTTGACATTGAACATCGTGCTGCATGGCTGCTCGCCAAACAAGAGCGCAACGGGTTCCCGTTTGACACAAAAGCAATCGAAGAGTTGTACGTAGAGTTACCTTCTCGTCGct                                                                                                                                                                                                                                                                                                  >  1:218055/1‑301 (MQ=255)`  
       `ggTGAAGAATACGTTGACGGAATGGAGTGGTGGAACTTCAACGAAGAGATGATGGACTATAACGTTCAGGACGTTGTGGTAACTAAAGCTCTCCTTGAGAAGCTACTCTCTGACAAACATTACTTCCCTCCTGAGATTGACTTTACGGACGTAGGATACACTACGTTCTGGTCAGAATCCCTTGAGGCCGTTGACATTGAACATCGTGCTGCATGGCTGCTCGCTAAACAAGAGCGCAACGGGTTCCCGTTTGACACAAAAGCAATCGAAGAGTTGTACGTAGGGTTAGCTTCTcgccgcc                                                                                                                                                                                                                                                                                                  >  1:431028/1‑300 (MQ=255)`  
       `ggTGAAGAATACGTTGACGGAATGGAGTGGTGGAACTTCAACGAAGAGATGATGGACTATAACGTTCAGGACGTTGTGGTAACTAAAGCTCTCCTTGAGAAGCTACTCTCTGACAAACATTACTTCCCTCCTGAGATTGACTTTACGGACGTAGGATACACTACGTTCTGGTCAGAATCCCTTGAGGCCGTTGACATTGAACATCGTGCTGCATGGCTGCTCGCTAAACAAGAGCGCAACGGGTTCCCGTTTGACACAAAAGCAATCGAAGAGTTGTAAGTAGAGTTAGCTTCTCGCCGct                                                                                                                                                                                                                                                                                                  >  1:199291/1‑301 (MQ=255)`  
          `gaagaaTACGTTGACGGAATGGAGTGGTGGAACTTCAACGAAGAGATGATGGACTATAACGTTCAGGACGTTGTGGTAACTAAAGCTCTCCTTGAGAAGCTACTCTCTGACAAACATTACTTCCCTCCTGAGATTGACTTTACGGACGTAGGATACACTACGTTCTGGTCAGAATCCCTTGAGGCCGTTGACATTGAACATCGTGCTGCATGGCTGCTCGCTAAACAAGAGCGCAACGGGTTCCCGTTTGACACAAAAGCAATCGAAGAGTTGAACGTAGAGTTAGCTTCTCGCCGCTCTg                                                                                                                                                                                                                                                                                               >  1:443613/1‑301 (MQ=255)`  
                `tACGTTGACGGAATGGAGTGGTGGAATGTCAACGAAGAGATGATGGACTATAACGTTCAGGACGTTGTGGTAACTAAAGCTCTCCTTGAGAAGCTACTCTCTGACATACAGTACTTCCCTCCTGAGATTGACTTTACGGACGTAGGTTACACTACGTTCTGGTCAGAATCCCTTGAGGCCGTTGACATTGAACATCGTGCTGCATGGCTGCTCGCTAAACAAGAGCGCAACGGGTTCCCGTTTGACACAAAAGCAATCGAAGAGTTGTACGTAGAGTTAGCTTCTCGCCGCTCTGAGTTGc                                                                                                                                                                                                                                                                                         <  1:1948/301‑1 (MQ=255)`  
                  `cGTTGACGGAATGTAGTGGTGGAACTTCAACGAAGAGATGATGGACTATAACGTTCAGGACGTTGTGGTAACTAAAGCTCTCCTTGAGAAGCTACTCTCTGACAAACATTACTTCCCTCCTGAGATTGACTTTACGGACGTAGGATACACTACGTTCTGGTCAGAATCCCTTGAGGCCGTTGACATTGAACATCGTGCTGCATGGCTGCTCGCTAAACAAGAGCGCAACGGGTTCCCGTTTGACACAAAAGCAATCGAAGAGTTGTACGTAGAGTTAGCTTCTCGCCGCTCTGAGTTGCTc                                                                                                                                                                                                                                                                                       <  1:343831/301‑1 (MQ=255)`  
                  `agTTGACGGAATGGAGTGGTGGAACTTCAACGAAGAGATGATGGACTATAACGTTCAGGACGTTGTGGTAACTAAAGCTCTCCTTGAGAAGCTACTCTCTGACAAACATTACTTCCCTCCTGAGATTGACTTTACGGACGTAGGATACACTACGTTCTGGTCAGAATCCCTTGAGGCCGTTGACATTGAACATCGTGCTGCATGGCTGCTCGCTAAACAAGAGCGCAACGGGTTCCCGTTTGACACAAAAGCAATCGAAGAGTTGTACGTAGAGTTAGCTTCTCGCCGCTCTGAGTTGCTc                                                                                                                                                                                                                                                                                       <  1:508694/300‑1 (MQ=255)`  
                              `ggAGTGGTGGAACTTCAACGAAGAGATGATGGACTATAACGTTCAGGACGTTGTGGTAACTAAAGCTCTCCTTGAGAAGCTACTCTCTGACAAACATTACTTCCCTCCTGAGATTGACTTTACGGACGTAGGATACACTACGTTCTGGTCAGAATCCCTTGAGGCCGTTGACATTGAACATCGTGCTGCATGGCTGCTCGCTAAACAAGAGCGCAACGGGTTCCCGTTTGACACAAAAGCAATCGAAGAGTTGTACGTAGAGTTATCTTCTCGACGCTCTGAGTTGCTCCGTCAATTGAcc                                                                                                                                                                                                                                                                           >  1:122836/1‑301 (MQ=255)`  
                               `gAGTGGTGGAACTTCAACGAAGAGATGATGGACTATAACGTTCAGGACGTTGTGGTAACTAAAGCTCTCCTTGAGAAGCTACTCTCTGACAAACATTACTTCCCTCCTGAGATTGACTTTACGGACGTAGGATACACTACGTTCTGGTCAGAATCCCTTGAGGCCGTTGTCATTGAACATCGTGCTGCATGGCTGCTCGCTAAACAAGAGCGCAACGGGTTCCCGTTTGACACAACAGCAATCGAAGAGTTGTACGTAGAGTTAGCTTCTCGCCGCTCTGAGTTGCTCCGTAAATTGACCg                                                                                                                                                                                                                                                                          >  1:243196/1‑301 (MQ=255)`  
                                 `gtggtgGAACTTCAACGAAGAGATGATGGACTATAACGTTCAGGACGTTGTGGTAACTAAAGCTCTCCTTGAGAAGCTACTCTCTGACAAACATTACTTCCCTCCTGAGATTGACTTTACGGACGTAGGATACACTACGTTCTGGTCAGAATCCCTTGAGGCCGTTGACATTGAACATCGTGCTGCATGGCTGCTCGCTAAACAAGAGCGCAACGGGTTCCCGTTTGACACAAAAGCAATCGAAGAGTTGTACGTAGAGTTAGCTTCTCGCCGCTCTGAGTTGCTCCGTAAATTGACCGaa                                                                                                                                                                                                                                                                        >  1:365660/1‑301 (MQ=255)`  
                                           `ttCAACGAAGAGATGATGGACTATAACGTTCAGGACGTTGTGGTAACTAAAGCTCTCCTTGAGAAGCTACTCTCTGACAAACATTACTTCCCTCCTGAGATTGACTTTACGGACGTAGGATACACTACGTTCTGGTCAGAATCCCTTGAGGCCGTTGACATTGAACATCGTGCTGCATGGCTGCTCGCTAAACAAGAGCGCAACGGGTTCCCGTTTGACACAAAAGCAATCGAAGAGTTGTACGTAGAGTTAGCTTCTCGCCGCTCTGAGTTGCTACGTAAATTGACCGAAACGTTCGGCt                                                                                                                                                                                                                                                              <  1:26997/301‑1 (MQ=255)`  
                                                      `gatgatGGACTATAACGTTCAGGACGTTGTGGTAACTAAAGCTCTCCTTGAGAAGCTACTCTCTGACAAACATTACTTCCCTCCTGAGATTGACTTTACGGACGTAGGATACACTACGTTCTGGTCAGAATCCCTTGAGGCCGTTGACATTGAACATCGTGCTGCATGGCTGCTCGCTAAACAAGAGCGCAACGGGTTCCCGTTTGACACAAAAGCAATCGAAGAGTAGTACGTAGAGTTAGCTTCTCGCCGCTCTGAGTTGCTCCGTAAATTGACCGAAACGTTCGGCCCGTGGGATCAg                                                                                                                                                                                                                                                   >  1:190605/1‑301 (MQ=255)`  
                                                      `gatgatGGACTATAACGTTCAGGACGTTGTGGTAACTAAAGCTCTCCTTGAGAAGCTACTCTCTGACAAACATTACTTCCCTCCTGAGATTGACTTTACGGACGTAGGATACACTACGTTCTGGTAAGAATCCCTTGAGGCCGTTGACATTGAACATCGTGCTGCATGGCTGCTCGCTAAACAAGAGCGCAACGGGTTCCCGTTTGACACAAAAGCAATCGAAGAGTTGTACGTAGAGTTAGCTTCTCGCCGCTCTGAGTTGCTCCGTAAATTGACCGAAACGTTCGGCTCGTGGTATCAg                                                                                                                                                                                                                                                   >  1:228678/1‑301 (MQ=255)`  
                                                            `ggACTATAACGTTCAGGACGTTGTGGTAACTAAAGCTCTCCTTGAGAAGCTACTCTCTGACAAACATTACTTCCCTCCTGAGATTGACTTTACGGACGTAGGATACACTACGTTCTGGTCAGAATCCCTTGAGGCCGTTGACATTGAACATCGTGCTGCATGGCTGCTCGCTAACCAAGAGCGCAACGGGTTCCCGTTTGACACAAAAGCAATCGAAGAGTTGTACGTAGAGTTAGCTTCTCGCCACTCTGAGTTGCTCCGTAAATTGACCGAAACTTTCGGCTCGTGGTATCCGccctgt                                                                                                                                                                                                                                             >  1:287178/1‑297 (MQ=255)`  
                                                              `cagttaACGTTAAGGACGTTGTGGTAACTAAAGCGCTCCTTGAGAAGCTACTCGCTGACAAACATTACTTCCCTCCTGAGATTGTCTTTACGGACGTAGGATACACTACGTTCTGGTCAGAATCCCTTGAGGCCGTTGACATTGAACATCGTGCTGCATGGCTGCTCGCTAAACAAGAGCGCAACGGGTTCCCGTTTGACACAAAAGCAATCGAAGAGTTGTACGTAGAGTTAGCTTCTCGCCGCTCTGAGTTGCTCCGTAAATTGACCGAAACGTTCGGCTCGTGGTATCAGCCTAAAgg                                                                                                                                                                                                                                           <  1:81579/297‑1 (MQ=255)`  
                                                              `attggtaaaagagacgggTTGTGGTAACTAAAGTTCTCCTTGAGAAGTTACTCTCTGACCAAAATTACTTCCCTCCTGGAATTGACTTTACGGAAGTAGGATACACTACGTTCTGGTCATAATCCCTTGAGGCCGTTGACATTGAACATCGTGCTGCATGGCTGCTCGCTAAACAAGAGCGCAACGGGTTCCCGTTTGACACAAAAGCAATCGAAGAGTTGTACGTAGAGTTAGCTTCTCGCCGCTCTGAGTTGCTCCATAAATTGACCGAAACGTTCGGCTCGTGGTATCAGCCTAAAgg                                                                                                                                                                                                                                           <  1:316786/284‑1 (MQ=255)`  
                                                              `agagagtagggtattaaggagattgaaCTAAAGGTCTCCTTGACAAGCTTATATCTGCCAAACATTACCTCTCTACTGAGATTGACCCGACGGACGCAGGATACCCTACGTTTTGGTCAGAATCTCGTGAGGCCGTTGACAATGCACATCGTGCTGCATGGCTGCTCGCTAAACAAGTGCGCAACGGGCTTCCGTTTTACACACAAGCAACCGAAGAGTTGTACGTAGAGTTAGCTGCGCGCCGCTCTGAGTTGCTCCGTACATTGACCGAAACGTTCGTCTCGTGGTATCAGCCTAAAgg                                                                                                                                                                                                                                           <  1:297534/276‑1 (MQ=255)`  
                                                              `aCTATAACGTTCAGGACGTTGTGGTAACTAAAGCTCTCCTTGAGAAGCTACTCTCTGACAAACATTACTTCCCTCCTGAGATTGACTTTACGGACGTAGGATACACTACGTTCTGGTCAGCATCCCTTGAGGCCGTTGACATTGAACATCGTGCTGCATGGCTGCTCGCTAAACAAGAGCGCAACGGGTTCCCGTTTGACACAAAAGCAATCGAAGAGTTGTACGTAGAGTTAGCTTCTCGCCGCTCTGAGTTGCTCCGTAAATTGACCGACACGTTCGGCCCGTGGTAGCAGCCTAAAgg                                                                                                                                                                                                                                           >  1:282265/1‑301 (MQ=255)`  
                                                              `aCTATAACGTTCAGGACGTTGTGGTAACTAAAGCTCTCCTTGAGAAGCTACTCTCTGACAAACATTACTTCCCTCCTGAGATTGACTTTACGGACGTAGGATACACTACGTTCTGGTCAGAATCCCTTGAGGCCGTTGACATTGAACATCGTGCTGCATGGCTGCTCGCTAAACAAGAGCGCAACGGGTTCCCGTTTGACACAAAAGCAATCGAAGAGTTGTACGTAGAGTTAGCTTCTCGCCGCTCTGAGTTGCTCCGTAAATTGACCGAAACGTTCGGCTCGTGGTATCAGCCTAAAgg                                                                                                                                                                                                                                           <  1:139223/301‑1 (MQ=255)`  
                                                              `aCTATAACGTTCAGGACGTTGTGGTAACTAAAGCTCTCCTTGAGAAGCTACTATCTGACAAACATTACTTCCCTCCTGAGATTGACTTTACGGACGTAGGATACACTACGTTCTGGTCAGAATCCCTTGAGGCCGTTGACATTGAACATCGTGCTGCATGGCTGCTCGCTAAACAAGAGCGCAACGGGTTCCCGTTTGACACAAAAGCAATCGAAGAGTTGTACGTAGAGTTAGATTCTCGCCGCTCTGAGTTGCTCCGTAAATTGACCGAAACGTTCGGCTCGTGGTATCAGCCTAAAgg                                                                                                                                                                                                                                           <  1:224735/301‑1 (MQ=255)`  
                                                               `cTATAACGTTCAGGACGTTGTGGTAACTAAAGCTCTCCTTGAGAAGCTACTCTCTGACAAACATTACTTCCCTCCTGAGATTGACTTTACGGACGTAGGATACACTACGTTCTGGTCAGAATCCCTTGCGGCCGTTGACATTGAACATCGTGCTGCATGGCTGCTCGCTAAACAAGAGCGCAACGGGTTCCCGTTTGACACAAAAGCAATCGACGAGTTGTACGTAGAGTTAGCTTCTCGCCGCTCTGAGTTGCTCCGTAAATTGACCGAAACGTTCGGATCGTGGTATACGCCTAAAGGt                                                                                                                                                                                                                                          >  1:498203/1‑301 (MQ=255)`  
                                                                 `ataACGTTCAGGACGTTGTGGTAACTAAAGCTCTCCTTGAGAAGCTACTCTCTGACAAACATTACTTCCCTCCTGAGATTGACTTTACGGACGTAGGATACACTACGTTCTGGTCAGAATCCCTTGAGGCCGTTGACATTAAACATCGTGCTGCATGGCTGCTCGCTAAACAAGAGCGCAACGGGTTCCCGTTTGACACAAAGGCAATCGAAGAGTTGTACGTAGAGTTAGCTGCTCGCCGCTCTGAGTTGCTCCGTAACTTGACCGCAACGTTCGGCTCGTGGTATCAGCCTAAAggctg                                                                                                                                                                                                                                        >  1:293115/1‑298 (MQ=255)`  
                                                                      `gTTCAGGACGTTGTGGTAACTAAAGCTCTCCTTGAGAAGCTACTCTCTGACAAACATTACTTCCCTCCTGAGATTGACTTTACGGACGTAGGATACACTACGTTCTGGTCAGAATCCCTTGAGGCCGTTTACATTGAACATCGTGCTGCATGGCTGCTCGCTAAACAAGAGCGCAACGGGTTCCCGTTTGACACAAAAGCAATCGAAGAGTTGTACGTAGAGTTAGCTTCTCGCCGCTCTGAGTTGCTCCGTAAATTGACCGAAACGTTCGGCTCGTGGTATCAGCCTaaatgctgtttct                                                                                                                                                                                                                                   >  1:325470/1‑291 (MQ=255)`  
                                                                      `gTTCAGGACGTTGTGGTAACTAAAGCTCTCCTTGAGAAGCTACTCTCTGACAAACATTACTTCCCTCCTGAGATTGACTTTACGGACGTAGGATACACTACGTTCTGGTCAGAATCCCTTGAGGCCGTTGACATTGAACATCGTGCTGCATGGCTGCTCGCTAAACAAGAGCGCAACGGGTTCCCGTTTGACACAAAAGCAATCGAAGAGTTGTACGTAGAGTTAGCTTCTCGCCGCTCTGAGTTGCTCCGTAAATTGACAGAAACGTTCGGCTCGTGGTTTCAGCCTAAAggctgtctct                                                                                                                                                                                                                                   >  1:339289/1‑293 (MQ=255)`  
                                                                              `cGTTGTGGTAACTAAAGCTCTCCTTGAGAAGCTACTCTCTGACAAACATTACTTCCCTCCTGAGATTGACTTTACGGACGTAGGATACACTACGTTCTGGTCAGAATCCCTTGAGGCCGTTGACATTGAACATCGTGCTGCATGGCTGCTCGCTAAACAAGAGCGCAACGGGTTCCCGTTTGACACAAAAGCAATCGAAGAGTTGTACGTAGAGTTAGCTTCTCGCCGCTCTGAGTTGCTCCGTAATTTGACCGAAACTTTCGGCTCGTGGTATCACCCTaacgcctgtctcctatacaca                                                                                                                                                                                                                           >  1:113928/1‑282 (MQ=255)`  
                                                                                                   `ccTTGAGAAGCTACTCTCTGACAAACATTACTTCCCTCCTGAGATTGACTTTACGGACGTAGGATACACTACGTTCTGGTCAGAATCCCTTGAGGCCGTTGACATTGAACATCGTGCTGCATGGCTGCTCGCTAAACAAGAGCGCAACGGGTTCCCGTTTGACACAAAAGCAATCGAAGAGTTGTACGTAGAGTTAGCTTCTCGCCGCTCTGAGTTGCTCCTTAAATTGCCCGAAACGTTCGGCTCGTGGTATCAGCCTACAGGTGGCACTGCTATTTTCTGcctgtctcttatacacatt                                                                                                                                                                                                      >  1:187299/1‑284 (MQ=255)`  
                                                                                                    `cTTGAGAAGCTACTCTCTGACAAACATTACTTCCCTCCTGAGATTGACTTTACGGACGTAGGATACACTACGTTCTGGTCAGAATCCCTTGAGGCCGTTGACATTGAACATCGTGCTGCATGGCTGCTCGCTAAACAAGAGCGAAACGGGTTCCCGTTTGACACAAAAGCAATCGAAGAGTTGTACGTAGAGTTAGCTTCTCGCCGCTCTGAGTTGCTCCGTAAATTGACCGAAACGTTCGGCTCGTGGTCTCAGCCTAAAGGTGGCACTGAGATGTTCTGCAATCCGCGGACAGGTAAGc                                                                                                                                                                                                     >  1:261683/1‑301 (MQ=255)`  
                                                                                                       `gacatgCTACTCTCTGACAAACATTACTTCCCTCCTGAGATTGACTTTACGGACGTAGGATACACTACGTTCTGGTCAGAATCCCTTGAGGCCGTTGACATTGAACATCGTGCTGCATGGCTGCTCGCTAAACAAGAGCGCAACGGGTTCCCGTTTGACACAAAAGCAATCGAAGAGTTGTACGTAGAGTTAGCTTCTCGCCGCTCTGAGTTGCTCCGTAAATTGACCGAAACGTTCGGCTCGTGGTATCAGCCTAAAGGTGGCACTGAGATGTTCTGCCATCCGCGAACAGGTAAGCCAc                                                                                                                                                                                                  <  1:204366/296‑1 (MQ=255)`  
                                                                                                           `aGCTACTCTCTGACAAACATTACTTCCCTCCTGAGATTGACTTTCCGGACGTAGGATACACTACGTTCTGGTCAGAATCCCTTGAGGCCGTTGACATTGAACATCGTGCTGCATGGCTGCTCGCTAAACAAGAGCGCAACGGGTTCCCGTTTGACACAAAAGCAATCGAAGAGTTGTACGTAGAGTTAGCTTCTCGCCGCTCTGAGTTGCTCCGTAAATTGACCGAAACGTTCGGCTCGTGGTATCAGCCTAAAGGTGGCACTGAGATGTTCTGCCATCCGCGAACAGGTAAGCCACTAcc                                                                                                                                                                                              <  1:415106/301‑1 (MQ=255)`  
                                                                                                           `aGCTACTCTCTGACAAACATTACTTCCCTCCTGAGATTGACTTTACGGACGTAGGATACACTACGTTCTGGTCAGAATCCCTTGAGGCCGTTTACATTGAACATCGTGCTGCATGGCTGCTCGCTAAACAAGAGCGCAACGGGTTCCCGTTTGACACAAAAGCAATCGAAGAGTTGTACGTAGAGTTAGCTTCTCGCCGCTCTGAGTTGCTCCGTAAATTGACCTAAACGTTCGGCTCGTGGTATCAGCCTAAAGGTGGCACTGAGATGTTCTGCCATCCGCGAACAGGTAAGCCACTAcc                                                                                                                                                                                              <  1:473145/301‑1 (MQ=255)`  
                                                                                                               `aCTCTCTGACAAACATTACTTCCCTCCTGATATTGACTTTACGGACGTAGGATACACTACGTTCTGGTCAGAATCCCTTGAGGCCGTTTACATTGAACATCGTGCTGCATGGCTGCTCGCTAAACAAGAGCGCAACGGGTTCCCGTTTGACACAAAAGCAATCGAAGAGTTGTACGTAGAGTTAGCTTCTCTCCGCTCTGAGTTGCTCCGTAAATTGACCGAAACGTTCGGCTCGTGGTATCAGCCTAAAGGTGGCACTGAGATGTTCTGCCATCCGCGAACAGGTAAGCCACTACCTaaa                                                                                                                                                                                          >  1:3827/1‑301 (MQ=255)`  
                                                                                                                  `ctctGACAAACATTACTTCCCTCCTGAGTTTGACTATACGGACGTAGGATACACTACGTTCTGGTCAGAAGCCCTTGAGGCCGTTGACATTGAACATCGTGCTGCATTGCTGCTCGCTAAACAAGAGCGCAACGGGTTCCCGTTTGACACAAAAGCAATCGAAGAGTTGTACGTAGAGTTAGCTTCTCGCCGCTCGGAGTTGCTCCGTAAATTGACCGAAACGTTCGGCTCGTGGTATCAGCCTAAAGGTGGCACTGAGATGTTCTGCCATCCGCGAACAGGTAAGCCACTACATAAATAc                                                                                                                                                                                       <  1:275915/301‑1 (MQ=255)`  
                                                                                                                  `ctctGACAAACATTACTTCCCTCCTGAGATTGACTTTACGGACGTAGGATACACTACGTTCTGGTCAGAATCCCTTGAGGCCGTTGACATTGAACATCGTGCTGCATGGCTGCTCGCTAAACAAGAGCGCAACGGGTTCCCGTTTGACACAAAAGCAATCGAAGAGTTGTACGTAGAGTTAGCTTCTCGCCGCTCTGAGTTGCTCCGTAAATTGACCGAAACGTTCGGCTCGTGGTATCAGCCTAAAGGTGGCACTGAGATGTTCTGCCATCCGCGAACAGGTAAGCCACTACCTAAATAc                                                                                                                                                                                       <  1:135063/301‑1 (MQ=255)`  
                                                                                                                  `ctctGACAAACATTACTTCCCGCCTTGGATTGGCTTTACGGACGTAGGATACACTACGTTCTGGTCAGAAGCCCTTGAGGCCGTTGACATTGAACATTGTGCTGCATGGCTGCTCGCTAAACAAGAGCGCAACGGGTTCCCGTTTGACACAAAAGCAATCGAAGAGTTGTACGTAGAGTTAGCTTCTCGCCGCTCTGAGTTGCTCCGTAAATTGACCGAAACGTTCGGCTCGTGGTATCAGCCTAAAGGTGGCACTGAGATGTTCTGCCATCCGCGAACAGGTAAGCCACTACCTAAATAc                                                                                                                                                                                       <  1:396851/301‑1 (MQ=255)`  
                                                                                                                  `atctGACAAACATTACTTCCCTCCTGCGATTGACTTTACGGACGTAGGATACACTACGTTCTGGTCAGAATCCCTTGAGGCCGTTGACATTGAACATCGTGCTGCATGGCTGCTAGCTAAACAAGAGCGCAACGGGTTCCCGTATGACACAAAAGCAATCGAAGAGTTGTACGTAGAGTTAGCTTCTCGCCGATCTGAGTTGCTCCGTAAATTGACCGAAACGTTCGGCTCGTGGTATCAGCCTAAAGGTGGCACTGAGATGTTCTGCCATCCGCGAACAGGTAAGCCACTACCTAAATAc                                                                                                                                                                                       <  1:307681/300‑1 (MQ=255)`  
                                                                                                                  `acaggACAAACATTCCTTCCCTCCTGAGATTTACTTTACGGACGTAGGATACACTACGTGCTGGTCAGAATCCCTTGAGGCCGTTGACATTGAACATCGTGCTGCATGGCTGCTCGCTAAACAAGAGCGCAACGGGTTCCCGTTTGACACAAAAGCAATCGAAGAGTTGTACGTAGAGTTAGCTTCTCGCCGCTCTGAGTTGCTCCGTAAATTGACCGAAACGTTCGGCTCGTGGTATCAGCCTAAAGGTGGCACTGAGATGTTCTGCCATCCGCGAACAGGTAAGCCACTACCTAAATAc                                                                                                                                                                                       <  1:100035/297‑1 (MQ=255)`  
                                                                                                                    `gtgtataggagaacgtCCCTCCGGAGATTGACTTTACGGACGTAGGATACACTACGTTATGGTAAGAATCCCTTGAGGCCGTTGACATTGAACATCGTGCTGCATGGCTGCTCGCTAAACAAGAGCGCAACGGGTTCCCGTTTGACACAAAAGCAATCGAAGAGTTGTACGTAGAGTTAGCTTCTCGCCGCTCTGAGTTGCTCCGTAAATTGACCGAAACGTTCGGCTCGTGGTATCATCCTAAAGGTGGCACTGAGATGTTCTGCCATCCGCGAACAGGTAAGCCACTACCTAAATAccc                                                                                                                                                                                     <  1:349090/286‑1 (MQ=255)`  
                                                                                                                    `ctGACAAACATTACTTCCCTCCTGAGATTGACTTTACGGACGTAGGATACACTACGTTCTTGTCAGAATCCCTTGAGGCCGTTGACATTGAACATCGTGCTGCATGGCTGCTCGCTAAACAAGAGCGCAACGGGTTCCCGTTTGACACAAAAGCAATCGAAGAGTTGTACGTAGAGTTAGCTTCTCGCCGCTCTGAGTTGCTCCGTAAATTGACCGAAACGTTCGGCTCGTGGTATCAGCCTAAAGGTGGCACTGAGATGTTCTGCCATCCGCGAACAGGTAAGCCACTACCTAAATActt                                                                                                                                                                                     >  1:90346/1‑299 (MQ=255)`  
                                                                                                                          `aaCATTACTTCACTCCTGAGATTGACTTTACGGACGTAGGATACACTACGTTCTGGTCAGAATCCCTTGAGGCCGTTGACATTGAACATCGTGCTGCATGGCTGCTCGCTAAACAAGAGCGCAACGGGTTCCCGTTTGACACAAAAGCAATCGAAGAGTTGTACGTAGAGTTAGCTTCTCGCCGCTCTGAGTTGCTCCGTAAATTGACCGAAACGTTCGGCTCGTGGTATCAGCCTAAAGGTGGCACTGAGATGTTCTGCCATCCGCGAACAGGTAAGccctgtctcttatacacatctcc                                                                                                                                                                               >  1:480345/1‑280 (MQ=255)`  
                                                                                                                           `aCATTACTTCCCTCCTGAGATTGACTTTACGGACGTAGGATACACTACGTTCTGGTCAGAATCCCTTGAGGCCGTTGACATTGAACATCGTGCTGCATGGCTGCTCGCTAAACAAGAGCGCAACGGGTTCCAGTTTGACACAAAAGCAATCGAAGAGTTGTACGTAGAGTTAGCTTCTCGCCGCTCTGAGTTGCTCCGTAAATTGACCGAAACGTTCGGCTCGTGGTATCAGCCTAAAGGTGTCACTGAGATGTTCTGCCATCCGCGAACAGGTAAGCCACTACCTAAATActttctctta                                                                                                                                                                              >  1:86418/1‑292 (MQ=255)`  
                                                                                                                              `ttACTTCCCTCCTGAGATTGACTTTACGGACGTAGTATACACTACGTTCTGGTCAGAATCCCTTGAGGCCGTTGACATTGAACATCGTGCTGCATGGCTGCTCGCTAAACAAGAGCGCAACGGGTTCCCGTTTGACACAAAAGCAATGGAAGAGTTGTACGTAGAGTTAGCTTCTCGCCGCTCTGAGTTGCTCCGTAAATTGACCGAAACGTTCGGCTCGTGGTATCAGCCTAAAGGTGGCACTGAGATGTTCTGCCATCCGCGAACAGGTAAGCCACTACCTAAATACCCTCGCATGAAg                                                                                                                                                                           <  1:209050/301‑1 (MQ=255)`  
                                                                                                                              `ttACTTCCCTCCTGAGATTGACTTTACGGACGTAGGATACACTACGTTCTGGTCCGAATCCCTTGAGGCCGTTGACATTGAACATCGTGCTGCATGGCTGCTCGCTAAATAAGAGCGCAACGGCTTCCCGTTTGACACAAAAGCAATCGAAGAGTTGTACGTAGAGTTAGCTTCTCGCCGCTCTGAGTTGCTCCGTAAATTGACCGAAACGTTCGGCTCGTGGTATCAGCCTAAAGGTGGCACTGAGATGTTCTTCCATTCGCGAACAGGTAAGCCACTACCTAACTACCCTCTCATTaaa                                                                                                                                                                           >  1:352793/1‑300 (MQ=255)`  
                                                                                                                              `ttACTTCCCTCCTGAGATTGACTTTACGGACGTAGGATACACTACGTTCTGGTCAGAATCCCTTGAGGCCGTTGACATTGAACATCGTGCTGCATGGCTGCTCGCTAAACAAGAGCGCAACGGGTTCCCGTTTGACACAAAAGCAATCGAAGAGTTGTACGTAGAGTTAGCTTCTCGCCGCTCTGAGTTGCTCCGTAAATTGACCGAAACGTTCGGCTCGTGGTATCAGCCTAAAGGTGGCACTGAGATGTTCTGCCATCCGCGAACAGGTAAGCACCTACCTAAATAcctgtaccttata                                                                                                                                                                           >  1:486662/1‑290 (MQ=255)`  
                                                                                                                                `aCTTCCTTCCTGAGATTGACTTTACGGACGTAGGATACACTACGTTCTGGTCAGAATCCCTTGAGGCCGTTGACATTGAACATCGTGCTGCATGGCTGCTCGCTAAACAAGAGCGCAACGGGTTCCCGTTTGACACAAAAGCAATCGAAGAGTTGTACGTAGAGTTAGCTTCTCGCCGCTCTGAGTTGCTCCGTAAATTGACCGAAACGTTCGGCTCGTGGTATCAGCCTAAAGGTGGCACTGAGATGTTCTGCCATCCGCGAACAGGTAAGCCACTACCTAAATACCCTCGCATTAAGac                                                                                                                                                                         <  1:321569/301‑1 (MQ=255)`  
                                                                                                                                `aCTTCCCTCCTGAGATTGACTTGACGGACGCAGGATACACTACGTTCTGGTCAGAATCCCTTGATGCCGTTGACATTGAACCTCGTGCTTCATGGCTGCTCACTAAACAAGAGCGCAACGGGTTCACGTTTGACACAAAAGCAATCGAAGAGTTGTACGTAGAGTTAGCTTCTCGCCGCTCTGAGTTGCTCCGTAAATTGACCGAAACGTTCGGCTCGTGGTATCAGCCTAAAGGTGGCACTGAGATGTTCTGCCATCCGCGAACAGGTAAGCCACTACCTAAATACCCTCGCATTAAGac                                                                                                                                                                         <  1:247117/301‑1 (MQ=255)`  
                                                                                                                                 `cTTCCCTCCTGAGATTGACTTTACGGACGTAGGATACACTACGTTCTGGTCAGAATCCCTTGAGGCCGTTGACATTGAACATCGTGCTGCATGGCTGCTCGCTAAACAAGAGCGCCACGGGTTCCCTTTTGACACAAAAGCAATCGCAGAGTTGTACGTAGAGTTAGCTTCTCGCCGCTCTGAGTTGCTCCGTAAATTGACCTAAACGTTCGGCTCGTTGTATCAGCCTAACGGTGGCACTGAGATGTTCTGCCATCCGCGCACAGGTACGCCACTACCTACATACCCTCGCATTAAGaca                                                                                                                                                                        >  1:213943/1‑301 (MQ=255)`  
                                                                                                                                         `cggagatgtttataagagacagaGGATACACTACGTTCTGGTCAGAATCCCTTGAGGCCGTTGACATTGAACATCGTGCGGCATGGCTGCTCGCTAAACAAGAGCGCAACGGGTTCCCGTTTGACACAAAAGCAATCGAAGAGTTGTACGTAGAGTTAGCTTCTCGCCGCTCTGAGTTGCTCCGTAAATTGACCGAAACGTTCGGCTCGTGGTATCAGCCTAAAGGTGGCACTGAGATGTTCTGCCATCCGCGAACAGGTAAGCCACTACCTAAATACCCTCGCATTAAGACACCTAAAGt                                                                                                                                                                <  1:37375/279‑1 (MQ=255)`  
                                                                                                                                          `caggaTTGACTTTACGGACGTAGGATACACTACGTTCTGGTCAGAATCCCTTGAGGCCGTTGACATTGAACATCGTGCTGCATGGCTGCTCGCTAAACAAGAGCGCAACGGGTTCCCGTTTGACACAAAAGCAATCGAAGAGTTGTACGTAGAGTTAGCTTCTCGCCGCTCTGAGTTGCTCCGTAAATTGACCGAAACGTTCGGCTCGTGGTATCAGCCTAAAGGTGGCACTGAGATGTTCTGCCATCCGCGAACGGGTAAGCCACTACCTAAATACCCTCGCATTAAGACACCTAAAGtt                                                                                                                                                               <  1:179934/298‑1 (MQ=255)`  
                                                                                                                                           `gagaTGGACTTTACGGACGTAGGATACACTACGTTCTGGTCAGAATCCCTTGAGGCCGTTGACATTGAACATCGTGCTGCATGGCTGCTCGCTAAACAAGAGCGCAACGGGTTCCCGTTTGACACAAAAGCAATCGAAGAGTTGTACGTAGAGTTAGCTTCTCGCCGCTCTGAGTTGCTCCGTAAATTGACCGAAACGTTCGGCTCGTGGTATCAGCCTAAAGGTGGCACTGAGATGTTCTTACATCCGCGAACAGGTAAGCCAATACCTCAATACCCTCGCATTAAGACACCTAAAGTtg                                                                                                                                                              >  1:303248/1‑301 (MQ=255)`  
                                                                                                                                               `agacagttACGGAAGGAGGATACACTACGTTCTGGTAAGAATCCCTTGAGGCCGTTGACATTTAACATCGTGCTGCATGGCTGCTCGCTAAACAAGAGCGCAACGGGTTCCCGTTTGACACAAAAGCAATCGAAGAGGTGTACGTAGAGTTAGCTTCTCGCCGCTCTGAGTTGCTTCGTAAATTGACCGAAACGTTCGGCTAGTGGTATCAGCCTAAAGGTGGCACTGAGATGTTCTGCCATCCGCGAACAGGTAAGACACTACCTAAATACCCTCGCATTAAGACACCTAAAGTtggtgg                                                                                                                                                          <  1:57408/295‑1 (MQ=255)`  
                                                                                                                                                  `tgtataagagacagggATACACTACGTTCTGGTCAGAATCCCTGGAGGCCGTTGACATTGAACATCGTGCTGCATGGCTGCTCGCTAAACAAGAGCGCAACAGGTTCCCGTTTGACACAAAAGCAATCGAAGAGTTGTACGTAGAGTTAGCTTCTCGCCGCTCTGAGTTGCTCCGTAAATTGACCGAAACGTTCGGCTAGTGGTATCAGCCTAAAGGTGGCACTGAGATGTTCTGCAATCCGCGAACAGGTAAGCCACTACCTAAATACCCTCGCATTAAGACACCTAAAGTTGGTGGTAt                                                                                                                                                       <  1:169319/287‑1 (MQ=255)`  
                                                                                                                                                       `aCGGACGTAGGATACACTACGTTCTGGTCAGAATCCCTTGAGGCCGTTGACATTGAACATCGTGCTGCATGGCTGCTCGCTAAACAAGAGCGCAACGGGTTCCCGTTTGACACAAAAGCAATCGAAGAGTTGTACGTAGAGTTAGCTTCTCGCCGCTCTGAGTTGCTCCGTAAATTGACCGAAACGTTCGGCTCGTGGTATCAGCCTAAAGGTGGCACTGAGATGTTCTGCCATCCGCGAACAGGTAAGCCACTACCTAAATACCCTCGCATTAAGACACCTAAAGTTGGTGGTATCtgtc                                                                                                                                                  >  1:117919/1‑298 (MQ=255)`  
                                                                                                                                                         `ggACGTAGGATACACTACGTTCTGGTCAGAATCCCTTGAGGCCGTTGACATTGAACATCGTGCTGCATGGCTGCTCGCTAAACAAGAGCGCAACGGGTTCCCGTTTGACACAAAAGCAATCGAAGAGTTGTACGCAGAGTTAGCTTCTCGCCGCTCTGAGTTGCTCCGTAAATTGACCGAAACGTTCGGCTCGTGGTATCAGCCTAAAGGTGGCACTGAGATGTTCTGCCATCCGCGAACAGGTAAGCCACTACCTAAATACCATCGCATTAAGACACCTAAAGTTGGTGGTATCTTTaag                                                                                                                                                <  1:14951/301‑1 (MQ=255)`  
                                                                                                                                                             `gTAGGATACACTACGTTCTGGTCAGAATCCCTTGAGGCCGTTGACATTGAACATCGTGCTGCATGGCTGCTCGCTAAACAAGAGCGCAACGGGTTCCCGATTGACACAAAAGCAATCGAAGAGTTGTACGTAGAGTTAGCTTCTCGCCGCTCTGAGTTGCTCCGTAAATTGACCGAAACGTTCGGCTCGTGGTATCAGCCTAAATGTGGCACTGAGATGTTCTGCCATCCGCGAACAGGTAAGCCACTACCTAAATACCCTCGCATTAAGACACCTAAAGTTGGTGGTATCTTTAAGAAGc                                                                                                                                            >  1:109169/1‑301 (MQ=255)`  
                                                                                                                                                               `aGGATACACTACGTTCTGGTCAGAATCCCTTGAGGCCGTTGACATTGAACATCGTGCTGCATGGCTGCTCGCTAAACAAGAGCGCAACGGGTTCCAGTTTGACACAAAAGCAATCGAAGAGTTGTACGTAGAGTTAGCTTCTCGCCGCTCTGAGTTGCTCCGTAAATTGACCGAAACGTTCGGCTCGTGGTATCAGCCTAAAGGTGGCACTGATATGTTCTGCCATCCGCGAACAGGTACGCCACTACCTTAATACCCTCGCATTAAGACACCTAAAGTTGGTGGTATCTTTAAGAAGCCt                                                                                                                                          >  1:154540/1‑301 (MQ=255)`  
                                                                                                                                                                `ggATACACTACGTTCTGGTCAGAATCCCTTGAGGCCGTTGACATTGAACATCGTGCTGCATGGCTGCTCGCTAAACAAGAGTGCAACGGGTTCCCGTTTGACACAAAAGCAATCGAAGAGTTGTACGTAGAGTTAGCTTCTCGCCGCTCTGAGTTGCTCCGTAAATTGACCGAAACGTTCGGCTCGTGGTATCAGCCTAAAGGTGGCACTGAGATGTTCTTCCATCCGCGAACAGGTAAGCCACTACCTAAATACCCTCGCATTAAGACAACTAAAGTTGGTGGTATCTTTAAGCAGCCTa                                                                                                                                         >  1:422126/1‑301 (MQ=255)`  
                                                                                                                                                                `ggATACACTACGTTCTGGTCAGAATCCCTTGAGGCCGTTGACATTGAACATCGTGCTGCATGGCTGCTCGCTAAACAAGAGCGCAACGGGTTCCCGTTTGACACAAAAGCAATCGAAGAGTTGTACGTAGAGTTAGCTTCTCGCCGCTCTGAGTTGCTCCGTAAATTGACCGAAACGTTCGGCTCGTGGTATCAGCCTAAAGGTGGCACTGAGATGTTCTGCCATCCGCGAACAGGTAAGCCACTACCTAAATACCCTCGCATTAAGACACCTAAAGTTGGTGGTATCTTTAAGAAGCCTa                                                                                                                                         >  1:377545/1‑301 (MQ=255)`  
                                                                                                                                                                 `gATACACTACGTTCTGGTCAGAATCCCTTGAGGCCGTTGACATTGAACATCGTGCTGCATGGCTGCTCGCTAAACAAGAGCGCAACGGGTTCCCGTTTGACACAAAAGCAATCGAAGAGTTGTACGTAGAGTTAGCTTCTCGCCGCTCTGAGTTGCTCCGTAAATTGACCGAAACGTTCGGCTCGTGGTATCAGCCTAAAGGTGGCACTGAGATGTTCTGCCATCCGCGAACAGGTAAGCCACTACCTAAATACCCTCGCATTAAGACACCTAAAGTTGGTGGTATCTTTAAGAAGCCTaa                                                                                                                                        >  1:233646/1‑301 (MQ=255)`  
                                                                                                                                                                  `aTACACTACGTTCTGGTCAGAATCCCTTGAGGCCGTTGACATTGAACATCGTGCTGCATGGCTGCTAGCTAAACAAGAGCGCAACGGGTTCCCGTTTGACACAAAAGCAATCGAAGAGTTGTACGTAGAGTTAGCTTCTCGCCGCTCTGAGTTGCTCCGTAAATTGACCGAAACGTTCGGCTCGTGGTATCAGCCTAAAGGTGGCACTGAGATGTTCTGCCATCCGCGAACAGGTAAGCCACTACCTAAATACCCTCGCATTAAGACACCTAAAGTTGGTGGTATCTTTAAGAAGCCTAAg                                                                                                                                       >  1:184000/1‑301 (MQ=255)`  
                                                                                                                                                                     `cacTACGTTCTGGTCAGAATCCCTTGAGGCCGTTGACATTGAACCTCGTGCTGCATGGCTTCTCGCTAAACAAGAGCGCTACGGGTTCCCGTTTGACACAAAAGCAATCGAAGAGTTGTACGTAGAGTTAGCTTCTCGCCGCTCTGAGTTGCTCCGTAAATTGACCGAAACGTTCGGCTCGTGGTATCAGCCTAAAGGTGGCACTGAGATGTTCTGCCATCCGCGAACAGGTAAGCCACTACCTAAATACCCTCGCATTAAGACACCTAAAGTTGGTGGTATCTTTAAGAAGCCTAAGAAc                                                                                                                                    <  1:104313/301‑1 (MQ=255)`  
                                                                                                                                                                     `cacTACGTTCTGGTCAGAATCCCTTGAGGCCGTTGACATTGAACATCGTGCTGCATGGCTGCTCGCTAAACAAGAGCGCAACGGGTTCCCGTTTGACACAAAAGCAATCGAAGAGTTGTACGTAGAGTTAGCTTCTCGCCGCTCTTAGTTGCTCCGTAAATTGACCGAAACGTTCGGCTCGTGGTATCAGCCTAAAGGTGGCACTGAGATGTTCTGCCATCCGCGAACAGGTAAGCCACTACCTAAATACCCTCGCATTAAGACACCTAAAGTTGGTGGTATCTTTAAGAAGCCTAAGaaa                                                                                                                                    >  1:493036/1‑300 (MQ=255)`  
                                                                                                                                                                     `cacTACGTTCTGGTAAGAATCCCTTGAGGCCGTTGACATTGAACATCGTGCTGCATGGCTGCTCGCTAAACAAGAGCGCAACGGGTTCCCGTTTGACACAAAAGCAATCGAAGAGTTGTACGTAGAGTTAGCTTCTCGCCGCTCTGAGTTGCTCCGTAAATTGACCGAAACGTTCGGCTCGTGGTATCAGCCTAAAGGTGGCACTGAGATGTTCTGCCATCCGCGAACAGGTAAGCCACTACCTAAATACCCTCGCATTAAGACACCTAAAGTTGGTGGTATCTTTAAGAAGCCTAAGAAc                                                                                                                                    <  1:130516/301‑1 (MQ=255)`  
                                                                                                                                                                     `acagaCGTTCTGGTCAGAATCCCTTGAGGCCGTTGACATTGAACATCGTGCTGCATGGCTGCTCGCGAAACAAGAGCGCAACGGGTTCCCGTTTGACACAAAAGCAATCGAAGAGTTGTACGTAGAGTTAGCTTCTCGCCGCTCTGAGTTGCTCCGTAAATTGACCGAAACGTTCGGCTCGTGGTATCAGCCTAAAGGTGGCACTGAGATGTTCTGCCATCCGCGAACAGGTAAGCCACTACCTAAATACCCTCGCATTAAGACACCTAAAGTTGGTGGTATCTTTAAGAAGCCTAAGAAc                                                                                                                                    <  1:455314/297‑1 (MQ=255)`  
                                                                                                                                                                      `acTACGTTCTGGTCAGAATCCCTTGAGGCCGTTGACATTGAACATCGTGCTGCATGGCTGCTCGCTAAACAAGAGCGCAACGGGTTCCCGTTTGACACAAAAGCAATCGAAGAGTTGTACGTAGAGTTAGCTTCTCGCCGCTCTGAGTTGCTCCGTAAATTGACCGAAACGTTCGGCTCGTGGTATCAGCCTAAAGGTGGCACTGAGATGTTCTGCCCTCCGCTAACAGGTAAGCCACTACCTAAATACCCTCGCATTAAGACACCTAAAGTTGGTGGTATCTTTAAGAAGCCTAAGactg                                                                                                                                   >  1:463744/1‑298 (MQ=255)`  
                                                                                                                                                                      `acTACGTTCTGGTCAGAATCCCTTGAGGCCGTTGACATTGAACATCGTGCTGCATGGCTGCTCGCTAAACAAGAGCGCAACGGGTTCCCGTTTGACACAAAAGCAATCGAAGAGTTGTACGTAGAGTTAGCTTCTCGCCGCTCTGAGTTGCTCCGTAAATTGACCGAAACGTTCGGCTCGTGGTATCAGCCTAAAGGTGGCACTGAGATGTTCTGCCATCCGCGAACAGGTAAGACACTACCTAAATACCCTCGCATTAAGACACCTAAAGTTGGTGGTATCTTTAAGAAGCCtgtctctt                                                                                                                                   >  1:173425/1‑294 (MQ=255)`  
                                                                                                                                                                        `tACGTTCTGGTCAGAATCCCTTGATGCCGTTGACATTGAACATCGTGCTGCATGGCTGCTCGCTAAACAAGAGCGCAACGGGTTCCCGTTTGACACAAAAGCAATCGAAGAGTTGTACGTAGAGTTAGCTTCTCGCCGCTCTGAGTTGCTCCGTAAATTGACCGAAACGTTCGGCTCGTGGTATCAGCCTAAAGTTGGCACTGAGATGTTCTGCCATCCGCGAACAGGTAAGCCACTACCTAAATACCCTCGCATTAAGACACCTAAAGTTGGTGGTATCTTTAAGAAGCCTAAGAACAAg                                                                                                                                 <  1:467756/301‑1 (MQ=255)`  
                                                                                                                                                                                `ggTCAGAATCCGTTGAGGCCGTTGACATTGAACATCTTTGTGCATGGCTGCTCGCTAAACAAGAGCGCAACGGGTTCCCGTTTTATACAAAAGCAATCGAATAGTTGTACGTAGAGTTAGCGTCTCGCCGATCTGAGTTGCTCCGTAAATTTACCGAAACGTTCGTCTCGTGGTATCAGCCTAAAGGTGGCACTGAGATGTTCTGCCATCCGCGAACAGGTAAGCCACTACCTAAATACCCTCGCATTAAGACACCTAAAGTTGGTGGTATCTTTAAGAAGCCTAATAACAAGGCACAGCg                                                                                                                         <  1:256258/301‑1 (MQ=255)`  
                                                                                                                                                                                `ggTCAGAATCCCTTGAGGCCGTTGACATTGAACATCGTGCTGCATGGCTGCTCGCTAAACAAGAGCGCAACGGGTTCCCGTTTGACACAAAAGCAATCGAAGAGTTGTACGTAGAGTTAGCTTCTCGCCGCACTGAGTTGCTCCGTAAATTGACCGAAACGTTCGGCTCGTGGTATCAGCCTAAAGGTGGCACTGAGATGTTCTGCCATCCGCGAAAAGGTAAGCTACTACCTAAATACCCTCGCATTAAGACACCTAAGGTTGGTGGTATCTTTAAGAAGCTTAAGAACAAGGCACAGCg                                                                                                                         >  1:16023/1‑301 (MQ=255)`  
                                                                                                                                                                                     `gAATCCCTTGAGTCCGTTGACATTGAACATCGTGCTGCATGGCTGCTCGCTAAACAAGAGCGCAACGGGTTCCCGTTTGACACAAAAGCAATCGAAGAGTTGTACGTAGAGTTAGCTTCTCGCCGCTCTGAGTTGCTCCGTAAATTGACCGAAACGTTCGGCTCGTGGTATCAGCCTAAAGGTGGCACTGAGATGTTCTGCCATCCGCGAACAGGTAAGCCACTACCTAAATACCCTCGCATTAAGACACCTAAAGTTGGTGGTATCTTTAAGAAGCCTAAGAACAAGGCACAGCGAGAAg                                                                                                                    <  1:329600/301‑1 (MQ=255)`  
                                                                                                                                                                                      `aaTCCCTTGAGGCCGTTGACATTGAACATAGTGCTGCATGGCTGCTCGCTAAACAAGAGCGCAACGGGTTCCCGTTTGACACAAAAGCAATCGAAGAGTTGTACGTAGAGTTAGCTTCTCGCCGCTCTGAGTTGCTACGTAAATTGACCGAAAGGTTCGGCTCGTGGTATCAGCCTAAAGGTGGCACTGAGATGTTCTGCCATCCGCGAACAGGTAAGCCACTACCTAAATACCCTCGCATTAAGACACCTAAAGTTGGTGGTATCTTTAAGAAGCCTAAGAACAAGGCACAGCGAGAAgg                                                                                                                   <  1:178189/301‑1 (MQ=255)`  
                                                                                                                                                                                       `aTCCCTTGAGGCCGTTGACATTGAACCTCGTGCTGAATAGTTGCTAGCTAAACAAGAGCGCAACGGGTTTCCGTTTGACACAAAAACCATCGAATAGTTGTACGTAGAGTTAGCTTCTCGCCGCTCTGAGTTGCTCCGTAAATTGACCGAAACGTTCGGCTCGTGGTATCAGCCTAAAGGTGGCACTGAGATGTTCTGCCCTCCGCGAACAGGTAAGCCACTACCTAAATCCCCTCGCATTAAGACACCTAAAGTTGGTGGTATCTTTAAGAAGCCTAAGAACAAGGCACAGCGAGAAGGc                                                                                                                  <  1:252083/301‑1 (MQ=255)`  
                                                                                                                                                                                         `cccTTGAGGCCGTTGACATTGAACATCGTGCTGCATGGCTGCTCGCTAAACAAGAGCGCAACGGGTTCCCTTTTGACACAAAAGCAATCGAAGAGTTGTACGTAGAGTTAGCTTCTCGCCGCTCTGAGTTGCTCCGTCAATTGACCGACACGTTCGGCTCGTGGTATCAGCCTAAAGGTGGCACTGAGATGTTCTGCCATCCGCGAACATGTAAGCCACTACCTAAATACCCTCGCCTTCAGACACCTAAAGTTGGTGGTATCTTTAAGAAGCCTAAGAACAAGGCCACGCGAGAAGGCCg                                                                                                                >  1:77194/1‑301 (MQ=255)`  
                                                                                                                                                                                          `ccTTGAGGCCGTTGACATTGAACATCGTGCTGCATAGCTGCTCGCTAAACAAGAGCGCAACGGGTTCCCGTTTGACACAAAAGCAATCGAAGAGTTTTACGTAGAGTTAGCTTCTCGACGCTCTGAGTTGCTCCGTAAATTGACCGAAACGTTCGGCTCGTGGTATCAGCCTAAAGGTGGCACTGAGATGTTCTGCCATCCTCGAACAGGTAAGCCACTACCTAAATACCCTCGCATTAAGACACCTCAAGTTGGTGGTCTCTTTAAGAAGCCTAAGAACAAGACACAGCGAGAAGGCCGt                                                                                                               >  1:265573/1‑301 (MQ=255)`  
                                                                                                                                                                                                       `gACATTGAACATCGTGCTGCATGGCTGCTCGCTAAACAAGAGCGCAACGGGTTCCCGTTTGACACAAAAGCAATCGAAGAGTTGTACGTAGAGTTAGCTTCTCGCCGCTCTGAGTTGCTCCGTAAATTGACCGAAACGTTAGGCTCGTGGTATCATCCTAAAGGTGGCACTGAGATGTTCTGCCATCCGCGAACAGGTAAGCCACTACCTAAATACCCTCGCATTAAGACACCTAAAGTTGGTGGTATCTTTAAGAAGCCTAAGAACAAGTCACAGCGAGACGGCCGTGAGACTTGCGAAc                                                                                                  >  1:370065/1‑301 (MQ=255)`  
                                                                                                                                                                                                        `aCATTGAACATCGTGCTGCATGGCTGCTCGCTAAACAAGAGCGCAACGGGTTCCCGTTTGACACAAAAGCAATCGAAGAGTTGTACGTAGAGTTAGCTTCTCGCCGCTCTGAGTTGCTCCGTAAATTGACCGAAACGTTCGGCTCGTGGTATCAGCCTAAAGGTGGCACTGAGATGTTCTGCCATCCGCGAACAGGTAAGCCACTACCTAAATACCCTCGCATTAAGACACCTAAAGTTGGTGGTATCTTTAAGAAGCCTAAGAACAAGGCACAGCGAGAAGGCCGTGAGCCTTGCGAAcc                                                                                                 >  1:440260/1‑300 (MQ=255)`  
                                                                                                                                                                                                        `aCATTGAACATCGTGCTGCATGGCTGCTCGCTAAACAAGAGCGCAACGGGTTCCCGTTTGACACAAAAGCAATCGAAGAGTTGTACGTAGAGTTAGCTTCTCGCCGCTCTGAGTTGCTCCGTAAATTAACCGAAACGTTCGGCTCGTGGTATCAGCCTAAAGGTGGCACTGAGATGTTCTGCCATCCGCGAACAGGTAAGCCACTACCTAAATACCCTCGCATTAAGACACCTAAAGTTGGTGGTATCTTTAAGAAGCCTAAGAAAAAGGCACAGCGTGAAGGCCGTGAGCCtctgtcttt                                                                                                 >  1:68166/1‑293 (MQ=255)`  
                                                                                                                                                                                                        `aCATTGAACATCGTGCTGCATGGCTGCTCGCTAAACAAGAGCGCAACGGGTTCCCGTTTGACACAAAAGCAATCAAAGAGTTGTACGTAGAGTTAGCTTCTCGCCTCTCTGAGTTGCTCCGTAAATTGACCGAAACGTTCGGCTCGTGGTATCAGCCTAAAGGTGGCACTGAGATGTTCTGCCATCCGCGAACAGGTAAGCCACTACCTAAATACCCTCGCATTAAGACACCTAAAGTTGGTGGTATCTTTAAGAAGCCTAAGAACAAGGCACAGCGAGAAGGCCGTGAGCCTTGCGAACt                                                                                                 >  1:40526/1‑301 (MQ=255)`  
                                                                                                                                                                                                        `aCATTGAACATCGTGCTGCATGGCTACTCGCTAAACAAGAGCGCAACGGGTTCCCGTTTGACACAAAAGCAATCGAAGAGTTGTACGTAGAGTTAGCTTCTCGCCGCTCTGAGTTGCTCCGTTAATTGACCGAAACGTTCGGCTCGTGGTATCAGCCTAAAGGTGGCCCTGAGATGTTCTGCCATCCGCGAACAGGTAAGCCACTACCTAAATACCCTCGCATTAAGACACCTAAAGTTGGTGGTATCTTTAAGAAGCCTAAGAACAAGGAACAGCGAGCAGGCCGTGAGCCTTGCGTACt                                                                                                 >  1:16525/1‑301 (MQ=255)`  
                                                                                                                                                                                                                              `ataagagacagaaCAAGAGCGCAACGGGTTCCCGTTTGACACAAAAGCAATCGAAGAGTTGTACGTAGAGTTAGCTTCTCGCCGCTCTGAGTTGCTCCGTAAATTGACCGAAACGTTCGGCTCGTGGTATCAGCCTAAAGGTGGCACTGAGATGTTCTGCCATCCGCGAACAGGTAAGCCACTACCTAAATACCCTCGCATTAAGACACCTAAAGTTGGTGGTATCTTTAAGAAGCCTAAGAACAAGGCACAGCGAGAAGGCCGTGAGCCTTGCGAACTTGATACCCGCGAGTACGTTGCt                                                                           <  1:435693/290‑1 (MQ=255)`  
                                                                                                                                                                                                                                   `tCGCTAAACAAGAGCGCAACGGGTTACCGTTTGACACAAAAGCAATCGAAGAGTTGTACGTAGAGTTAGCTTCTCACCGCTCTGAGTTGCTCCGTAAATTGACCGCAAAATTCGGCTCGTGGTCTCAGCCTAAAGATGGCACTGAGATGTTCTGCCATTCGCGAACAGGTAAGCAACTACCTAAATCCCCTCGAATTAAGACACCTAAAGTTGGTGGTGTCATTAAGAAGCCTAATAACAAGGCACAGCGAGAAGTCCGTGAGACTTGCGAACTTGATACGCACGCGTAGGGTGCTAGTgg                                                                      >  1:31211/1‑300 (MQ=255)`  
                                                                                                                                                                                                                                            `aagagacagaCGGGTTCCCGTTTGACACAAAAGCAATCGAAGAGTTGTACGTAGAGTTAGCTTCTCGCCGCTCTGAGTTACTCCGTAAATTGACCGAAACGTTCGGCTCGTGGTATCAGCCTAAAGGTGGCACTGAGATGTTCTGCCATCCGCGAACAGGTAAGCCACTACCTAAATACCCTCGCATTAAGACACCTAAAGTTGGTGGTATCTTTAAGAAGCCTAAGAACAAGGCACAGCGAGAAGGCCGTGAGCCTTGCGAACTTGATACCCGCGAGTACGTTGCTGGTGCTCCTTacac                                                             <  1:460170/292‑1 (MQ=255)`  
                                                                                                                                                                                                                                              `gagCGCATCGGGTTCCCGTTTGACACAAAAGCAATCGAAGAGTTGTACGTAGAGTTAGCTTCTCGCCGCTCTGAGTTGCTCCGTAAATTGACCGAAACGTTCGGCTCGTGGTATCAGCCTAAAGGTGGCACTGAGATGTTCTGCCATCCGCGAACAGGTAAGCCACTACCTAAATACCCTCGCATTAAGACACCTAAAGTTGGTGGTATCTTTAAGAAGCCTAAGAACAAGGCACAGCGAGAAGGCCGTGAGCCTTGCGAACTTGATACCCGCGAGTACGTTGCTGGTGCTCCTTACAccc                                                           >  1:334034/1‑301 (MQ=255)`  
                                                                                                                                                                                                                                                  `gcAACAGGTTCCCGCTTGACACAAAATCAATCCACGAGTTGTACGTAGATTTAGCTTCTCGCCGCTCTGAGTTGCTCCGTAAATTGACCGAAACGTTCGGCTCGTGGTATCAGCCTAAAGGTGGCACTGAGATGTTCTGCCATCCGCGAACAGGTAAGCCACTACCTAAATACCCTCGCATTAAGACACCTAAAGTTGGTGGTATCTTTAAGAAGCCTAAGAACAAGGCACAGCGAGAAGGCCGTGAGCCTTGCGAACTTGATACCCGCGAGTACGTTGCTGGTGCTCCTTACACCCCAGt                                                       <  1:53474/301‑1 (MQ=255)`  
                                                                                                                                                                                                                                                        `gggtcacGTTTGACACAAAAGCAATCGAAGAGTTGGACGTAGAGTGAGCTTCTCGCCGCTCTGAGGTGCTCCGTAAATTGACCGAAACGTTCGGATCGTGGTATCAGCATAAAGGTGGCACGGAGATGTTCTGCAATCCGAGAACAGGTAAGCCAATACCTAAATACCCTCGCATTAAGACACCTAAAGTTGGTGGTATCTTTAAGAAGCCTAAGAACAAGGCAAAGCGAGAAGGCCGTGAGCCTTGCGAACTTGATACCCGCGAGTACGTTGCTGGTGCTCCTTACACCCCAGTTGAACa                                                 <  1:144465/295‑1 (MQ=255)`  
                                                                                                                                                                                                                                                                    `acacAAAAGCAATCGAAGAGTTGTACGTAGAGTTAGCTTCTCGCCGCTCTGAGTTGCTCCGTAAATTGACCGAAACGTTCGGCTCGTGGTATCAGCCTAAAGGTGGCACTGAGATGTTCTGCCATCCGCGAACAGGTAAGCCACTACCTAAATACCCTCGCATTAAGACACCTAAAGTTGGTGGTATCTTTAAGAAGCCTAAGAACAAGGCACAGCGAGAAGGCCGTGAGCCTTGCGAACTTGATACCCGCGAGTACGTTGCTGGTGCTCCTTACACCCCAGTTGAACAtctgtctcttat                                     >  1:401517/1‑290 (MQ=255)`  
                                                                                                                                                                                                                                                                     `aacaAAAGCAATCGAAGAGTTGTACGTAGAGTTAGCTTCTCGCCGCTCTGAGTTGCTCCGTAAATTGACCGAAACGTTCGGCTCGTGGTATCAGCCTAAAGGTGGCACTGAGATGTTCTGCCATCCGCGAACAGGTAAGCCACTACCTAAATACCCTCGCATTAAGACACCTAAAGTTGGTGGTATCTTTAAGAAGCCTAAGAACAAGGCACAGCGAGAAGGCCGTGAGCCTTGCGAACTTGATACCCGCGAGTACGTTGCTGGTGCTCCTTACACCCCAGTTGAACATGTTGTGTTTAAc                                    <  1:361006/300‑1 (MQ=255)`  
                                                                                                                                                                                                                                                                       `agacaGCAATCGAAGAGTTGTACGTAGAGTTAGCTTCTCGCCGCTCTGAGTTGCTCCATAAATTGACCGAAACGTTCGGCTCGTGGTATCAGCCTAAAGGTGGCACTGAGATGTTCTGCCATCCGCGAACAGGTAAGCCACTACCTAAATACCCTCGCATTAAGACACCTAAAGTTGGTGGTATCTTTAAGAAGCCTAAGAACAAGGCACAGCGAGAAGGCCGTGAGCCTTGCGAACTTGATACCCGCGAGTACGTTGCTGGTGCTCCTTACACCCCAGTTGAACATGTTGTGTTTAAccc                                  <  1:426784/297‑1 (MQ=255)`  
                                                                                                                                                                                                                                                                             `cAATCGAAGAGTTGTACGTAGAGTTAGCTTCTCGACGCTCTGAGTTGCTCCGTAAATTGACCGAAACGTTCGGCTCGTGGTATCAGCCTAAAGGTGGCACTGAGATGTTCTGACATCCGCGAACAGGTAAGACACTACCTAAATACCCTCGCATTAAGACACCTAAAGTTGGTGGTATCTTTAAGAAGCCTAAGAACAAGGCACAGCGAGAAGGCCGTGAGCCTTGCGAACTTGATACCCGCGAGTACGTTGCTGGTGCTCCTTACACCCCAGTTGAACATGTTGTGTTTAACCCTTCGtc                            >  1:357245/1‑301 (MQ=255)`  
                                                                                                                                                                                                                                                                                     `gagTTGTACGTAGAGTTAGCTTCTCGCCGCTCTGAGTGGCTCCGTAAATTGACCGAAACGTTCGGCTCGTGGTATCAGCCTAAAGGTGGCACTGAGATGTTCTGCCATCCGCGAACAGGTAAGCCACTACTTAAATACCCTCGAATTAAGACACCTAAAGTTGGTGGGATCATTAAGAAGCCTAAGAACAAGGCACAGCGAGAAGGCCGTGAGCCTTGCGAACTTGATACCCGCGAGTACGTTGCTGGGGCTCCTTACACACCAGTTGAACATGTTGTGTTTAACCCTTCGACCCGTGAcc                    >  1:238636/1‑301 (MQ=255)`  
                                                                                                                                                                                                                                                                                       `gTTGTACGTAGAGTTAGCTTCTCGCCGCTCTGAGTTGCTCCGTAAATTGACCGAAACGTTCGGCTCGTGGTATCAGCCTAAAGGTGGCACTGAGATGTTCTGCCATCCGCGAACAGGTAATACAATATCTAAATACCCTCGCATTAAGACACCTAAAGTTGGTGGTATCTTTAAGAAGACTAAGAAAAAGGCACAGCGAGAAGGCCGTGCGCCTTGCGACCTTGATACCCGCGCGTACGTTGCTTGTGCTCCTTACTCCCCAGTTGCACTTATTGTGTTTAACCCTTCGTCTCGTGACcac                  >  1:94862/1‑301 (MQ=255)`  
                                                                                                                                                                                                                                                                                       `gTTGTACGTAGAGTTAGCTTCTCGCCGCTCTGAGTTGCTCCGTAAATTGACCGAAACGTTCGGCTCGTGGTATCAGCCTAAAGGTGGCACTGAGATGTTCTGCCATCCGCGAACAGGTAAGCCACTACCTAAATACCCTCGCATTAAGACACCTAAAGTTGGTGGTATCTTTAAGAAGCCTAAGAACAAGGCACAGCGAGAAGGACGTGAGCCTTGCGAACTTGATACCCGCGAGTACGTTGCTGGTGCTCCTTACACCCCAGTTGAACATGTTGTGTTTAACCCTTAGTCTCggaacaac                  >  1:62110/1‑294 (MQ=255)`  
                                                                                                                                                                                                                                                                                       `gTTGTACGTAGAGTTAGCTTCTCGCCGCTCTGAGTTGCTCCGTAAATTGACCGAAACGTTCGGCTCGTGGTATCAGCCTAAAGGTGGCACTGAGATGTTCTGCCATCCGCGAACAGGTAAGCCACTACATAAATACCCTCGCATTAAGACACCTAAAGTTGGTGGTATCTTTAAGAAGCCTAAGAACAAGGCACAGCGAGAAGGCCGTGAGCCTTGCGAACTTGATACCCGCGAGTACGTTGCTGGTGCTCCTTACACCCCAGTTGAACATGTTGTGTTTAACCCTTCGTCTCGTGGCcac                  >  1:476297/1‑301 (MQ=255)`  
                                                                                                                                                                                                                                                                                          `gTACGTAGAGTTAGCTTCTCGCCGCTCTGAGTTGCTCCGTAAATTGACCGAAACGTTCGGCTCGTGGTATCAGCCTAAAGGTGGCACTGAGATGTTCTGCCATCCGCGAACAGGTAAGCCACTACCTAAATACCCTCGCATTAAGACACCTAAAGTTGGTGGTATCTTTAAGAAGCCTAAGAACAAGGCACAGCGAGAAGGCCGTGAGCCTTTCGAACTTGATACCCGCGAGTACGTTGCTGGTGCTCCTTACACCCCAGTTGAACATGTTGTGTTTAACCCtgtctcttatacacatctc               >  1:386060/1‑283 (MQ=255)`  
                                                                                                                                                                                                                                                                                          `gTACGTAGAGTTAGCTTCTCGCCGCTCTGAGTTGCTCCGTAAATTGACCGAAACGTTCGGCTCGTGGTATCAGCCTAAAGGTGGCACTGAGATGTTCTGCCATCCGCGAACAGGTAAGCCACTACCTAAATACCCTCGCATTAAGACACCTAAAGTTGGTGGTATCTTTAAGAAGCCTAAGAACAAGGCACAGCGAGAAGGCCGTGAGCCTTGCGAACTTGATACCCGCGAGTACGTTGCTGGTGCTCCTTACACCCCAGTTGAACATGTTGTGTTTAACCCTTCGTCTCGTGACCACAtt               >  1:22195/1‑301 (MQ=255)`  
                                                                                                                                                                                                                                                                                          `gTACGTAGAGTTAGCTTCTCGCCGCTCTGAGTTGCTCCGTAAATTGACCGAAACGTTCGGATCGTGGTATCAGCCTAAAGGTGGAACTGAGATGTTCTGCCATCGGCGAACAGGTAAGCCACTACCTAAATACACTCGCATTAAGACAACTAAAGTTGGTGGTATCTTTAAGTAGCCTAAGAACAATGACAAGCGAGAAGGCAGTAAGCCTATCGAAATTTATCACAACGAGTACGTTGCTTGTGATCCTTCCACCCAAGTTGTAACTCTTGTGTTTAACCCTTCGTCCAGTGACCACCtt               >  1:504045/1‑301 (MQ=255)`  
                                                                                                                                                                                                                                                                                             `cGTAGAGTTAGCTTCTCGCCGCTCTGAGTTGCTCCGTAAATTGACCGAAACGTTCGGCTCGTGGTATCAGCCTAAAGGTGGCACTGAGATGTTCTGCCATCCGCGAACAGGTAAGCCACTACCTAAATACCCTCGCATTAAGACACCTAAAGTTGGTGGTATCTTTAAGAAGCCTAAGAACAAGGCACAGCGAGAAGGCCGTGAGCCTTGCGAACTTGATACCCGCGAGTACGTTGCTGGTGCTCCTTACACCCCAGTTGAACATGTTGTGTTTAACCCTTCGTCTCGTGACCACATTCag            >  1:410900/1‑301 (MQ=255)`  
                                                                                                                                                                                                                                                                                               `tAGAGTTAGCTTCTCGCCGCTCTGAGTTGCTCAGTAAATTGACCGAAACGTTCGGCTCGTGGTATCAGCCTAAAGGTGGCACTGAGATGTTCTGCCATCCGCGACCAGGTAAGCCACTACCTAAATACCCTCGCATTAAGACACCTACAGTTGGTGGTATCTTTAAGAAGCCTAAGAACAAGGCACAGCGAGAAGGCCGTGAGCCTTGCGAACTTGATACCCGCGAGTCCGTTGCTGGTTCTCCTTACACCCCAGTTGAACATGTTGTGTTTAACCCTTCGTCTCGTGACcacctgtctct          >  1:390558/1‑293 (MQ=255)`  
                                                                                                                                                                                                                                                                                                `agagTTAGCTTCTGTCCGCTCTGAGTTTCTCTGTAAATTGACCGATCCTTTCGGTTCGTGGTATCAGCCTAAAGGTGGCACTGAGATGTTCTGCCATCCGCGAACAGGTAAGCCACTACCTAAATACCCTCGCATTAAGACACCTAAAGTTGGGGTTATCTTTAAGAAGCCTAAGAACAAGGCACAGCTAGAAGGCCGTGAGCCTTGCGAACTTGATACCCGCGAGTACGTTGCTGTTTCTCCTTACACCCCAGTTTAACATGTTGTGTTTAACCCTTCGTCTCGTGACCACATTaagaag         <  1:44972/301‑1 (MQ=255)`  
                                                                                                                                                                                                                                                                                                   `gTTAGCTTCTCGCCGCTCTGAGTTGCTCCGTAAATTGACCGAAACGTTCGGATCGTGGTATCAGCCTAAAGGTGGCACTGAGATGTTCTGCCATCCGCGAACAGGTAAGCCACTACCTAAATACCCTCGCATTAAGACACCTAAAGTTGGTGGTATCTTTAAGAAGACTAAGAACAAGGAACAGCGAGAAGGCCGTGAGCCTTGCGAACTTGATACCCGCGAGTACGTTGCTGGTGCTCCTTACACCCCAGTTGAACATGTTGTGTTTAACCCTTCGTCTCGTGACCACATTCAGAAGaaa      >  1:127010/1‑301 (MQ=255)`  

                                                                                                                                                                                                                                                                                                          `|`                                                                                                                                                                                                                                                                                                           
`AGAGCAGGGTGAAGAATACGTTGACGGAATGGAGTGGTGGAACTTCAACGAAGAGATGATGGACTATAACGTTCAGGACGTTGTGGTAACTAAAGCTCTCCTTGAGAAGCTACTCTCTGACAAACATTACTTCCCTCCTGAGATTGACTTTACGGACGTAGGATACACTACGTTCTGGTCAGAATCCCTTGAGGCCGTTGACATTGAACATCGTGCTGCATGGCTGCTCGCTAAACAAGAGCGCAACGGGTTCCCGTTTGACACAAAAGCAATCGAAGAGTTGTACGTAGAGTTAGCTGCTCGCCGCTCTGAGTTGCTCCGTAAATTGACCGAAACGTTCGGCTCGTGGTATCAGCCTAAAGGTGGCACTGAGATGTTCTGCCATCCGCGAACAGGTAAGCCACTACCTAAATACCCTCGCATTAAGACACCTAAAGTTGGTGGTATCTTTAAGAAGCCTAAGAACAAGGCACAGCGAGAAGGCCGTGAGCCTTGCGAACTTGATACCCGCGAGTACGTTGCTGGTGCTCCTTACACCCCAGTTGAACATGTTGTGTTTAACCCTTCGTCTCGTGACCACATTCAGAAGAAACTCC  >  NC_001604.1/14796‑15391`

  

Alignment Legend

Aligned base mismatch/match (shaded by quality score): `ATCG/ATCG` `< 3 ≤` `ATCG/ATCG` `< 9 ≤` `ATCG/ATCG` `< 20 ≤` `ATCG/ATCG` `< 34 ≤` `ATCG/ATCG` `< 38 ≤` `ATCG/ATCG`

Unaligned base: `atcg`    Masked matching base: `atcg`    Alignment gap: `‑`    Deleted base: `‑`
